# Trait-related neural basis of attentional bias to emotions: a tDCS study

**DOI:** 10.3758/s13415-023-01122-3

**Published:** 2023-08-03

**Authors:** Angela Marotta, Miriam Braga, Mirta Fiorio

**Affiliations:** Department of Neurosciences, Biomedicine and Movement Sciences, University of Verona, Via Felice Casorati, 43-37131 Verona, Italy

**Keywords:** Negative affect, Attentional bias, Emotion, Prefrontal cortex, tDCS

## Abstract

Negative emotional stimuli can strongly bias attention, particularly in individuals with high levels of dispositional negative affect (NA). The current study investigated whether the prefrontal cortex (PFC), a brain region involved in the top-down regulation of emotional processing, plays a different role in controlling attention to emotions, depending on the individual NA. Sham and anodal transcranial direct current stimulation (tDCS) was delivered over the right or left PFC while assessing attentional bias (AB) to emotions (happy, angry, sad faces) in individuals with higher and lower trait NA. When tDCS was inactive (sham), individuals with higher trait NA showed AB toward angry and away from sad faces, while individuals with lower trait NA presented with no AB. Right anodal-tDCS abolished the AB toward angry faces and induced an AB toward sad faces in individuals with higher trait NA, while no effect was found in individuals with lower trait NA. Left anodal-tDCS abolished any AB in individuals with higher trait NA and induced an AB away from happy faces in individuals with lower trait NA. These findings confirm a critical role of trait NA in AB to emotions and demonstrate a different involvement of PFC in emotional processing based on dispositional affect.

## Introduction

In the daily life, our attention is constantly captured by salient stimuli, especially by those carrying emotional content. Attentional biases to emotions have an adaptive role; they help to regulate emotions, maintain an optimal homeostatic balance, and properly interact with other people. However, dysfunctional attentional biases to emotions can dramatically affect individual well-being. For instance, preferential and consistent attentional bias toward negative emotions is believed to contribute to the onset, development, and maintenance of many affective disorders (Bradley et al., 1998, 1999; Clarke et al., 2014; De Raedt & Koster, 2010; Joormann & Gotlib, 2007; MacLeod et al., 1986; Yiend, 2010).

Emerging evidence suggests that attentional bias to emotions can be influenced by personality traits; the most relevant is dispositional affect. This personality trait refers to a person’s stable tendency to engage in positive and negative moods, affect, and emotions (Watson & Clark, 1984).

Depending on dispositional affect, some individuals can be more prone than others to experience particular affective states. While individuals with high levels of dispositional negative affect are more prone to experience negative emotional states or mood, such as anger, anxiety, and sadness, along with poor self-concept, those with low levels of negative dispositional affect more frequently experience states of calmness and serenity. Conversely, individuals with high dispositional positive affect tend to feel enthusiastic, active, and alert, whereas those characterized by low levels of dispositional positive affect are more prone to experience sadness and lethargy (Garcia et al., 2015; Watson et al., 1988). Of note, dispositional affect can influence the way in which individuals respond to positive or negative emotional stimuli. With regards to this, converging lines of evidence suggest that dispositional negative affect in healthy individuals is associated with an attentional bias toward negative information, whereas dispositional positive affect determines attentional bias toward positive information, thus explaining away interindividual differences in attentional biases to emotions (Grafton & MacLeod, 2017; Lonigan & Vasey, 2009; Oehlberg et al., 2012; Onie & Most, 2017). Dispositional affect also has critical relevance in pathological conditions. For instance, a high level of dispositional negative affect is prevalent in individuals with affective disorders and also is considered a prospective risk factor for depression and anxiety (Böhnke et al., 2014; Joiner Jr. & Lonigan, 2000; Watson et al., 1988), whereas a high level of dispositional positive affect is associated with psychological well-being (Fredrickson, 2001). Taking this evidence together, an interplay emerges between dispositional affect and attentional biases to emotions, both in healthy and clinical populations.

The neural underpinnings of dispositional affect and attentional bias to emotions can be found in a functionally interactive network of cortical-limbic pathways that play a crucial role in emotion regulation (Banks et al., 2007; De Raedt et al., 2010; Phillips et al., 2008). While limbic areas (e.g., amygdala) are mainly involved in automatic engagement of attention to emotional information, the prefrontal cortex (PFC) is involved in the top-down control of attention via inhibitory pathways to limbic areas (Davidson & Irwin, 1999; Banks et al., 2007; Goldin et al., 2008; Notzon et al., 2018). Particularly, the PFC is considered a crucial hub for top-down attentional control, with a different involvement of the right and left hemispheres (Clarke et al., 2020; De Raedt et al., 2010; De Raedt & Koster, 2010; Ironside et al., 2019; Li et al., 2017; Sanchez-Lopez et al., 2018). Increased right PFC activation is associated with negative affect, mood/emotion dysregulation, and difficulties in attentional disengagement from negative emotions, while increased left PFC activation is associated with positive affect, effective emotional attention regulation, and attentional disengagement from negative emotional stimuli (Clarke et al., 2020; Davidson & Irwin, 1999; Ironside et al., 2019; Li et al., 2017; Sanchez-Lopez et al., 2018).

Whether the involvement of the right and the left PFC in attentional bias to emotions is influenced by dispositional affect is still unknown. By considering the evidence presented so far on the role of dispositional affect on attentional bias to emotions, and by considering the notion that personality traits influence the activation of brain regions involved in emotional processing (e.g., PFC) (Li et al., 2022; Peña-Gómez et al., 2011; Sagliano et al., 2016; Vanderhasselt et al., 2013), it is reasonable to hypothesize an interplay between dispositional affect, the PFC, and attentional bias to emotions.

The current study was designed to provide empirical evidence to this hypothesis by adopting Transcranial Direct Current Stimulation (tDCS), a neuromodulation technique in which two electrodes (anode and cathode) are mounted on the individual’s head to induce an electric current in the brain that can change neuronal excitability. In the case of anodal stimulation, the anode is positioned over the cortical site of interest and the cathode over a reference site (either cephalic or extracephalic), thus resulting in a depolarization of the resting membrane potential in the stimulated brain area. In the case of cathodal stimulation, the cathode is positioned over the cortical site of interest and the anode over the reference point, thus inducing a hyperpolarization in the stimulated brain area (Nitsche & Paulus, 2000). This technique has proven to be noninvasive and safe, with transient adverse effects (mainly in the form of tingling and itchiness on the stimulation site) of mild intensity (Bikson et al., 2016; Brunoni et al., 2011). To investigate for the first time whether dispositional affect influences the PFC in the top-down control of attentional bias to emotional stimuli, healthy participants were assessed for positive and negative dispositional affect and were asked to perform an emotional dot-probe task, a well-established paradigm to measure attentional bias to emotions. This task allows to distinguish two attentional processes, namely attendance (i.e., attentional bias toward emotional stimuli) and avoidance (i.e., attentional bias away from emotional stimuli) of emotions (Starzomska, 2017). During the task, tDCS was applied over the right or left DLPFC in different days.

We expected to find higher attentional bias toward negative emotions and lower attentional bias toward positive emotions in individuals with higher dispositional NA, while a reversed pattern of attentional bias was predicted in those with higher PA. With regards to the specific role of dispositional affect (negative vs. positive) in modulating the interplay between PFC and attentional bias to negative and positive emotions, our prediction is less straightforward, being this study the first to address this issue.

Understanding the psychological and neural determinants of attentional biases to emotions by considering the role of dispositional affect is of utmost importance for implementing effective and personalized interventions to improve individuals' abilities to regulate emotions.

## Materials and methods

### Participants

Because there is no published data on tDCS modulation of attentional bias to emotions depending on dispositional affect, the sample size was computed on *a priori* standard values by using G-Power 3 (Faul et al., 2007). We chose an anticipated effect size f of 0.25, which is suitable for within-between designs and is considered medium, according to the convention established by Cohen for estimating effect size in the absence of previous data (Cohen, 1988). Alpha error probability (or type I error rate) was set at 0.05, and power (1 − β error probability) at 0.90. Based on these parameters, the required sample size was 36. We recruited more participants to avoid a reduction of statistical power due to potential drop out. In all, 39 healthy participants with normal or corrected to normal vision volunteered for the study (18 women, mean age ± standard deviation 22.9 ± 3.6 years).

Before the experiment, participants were screened through an ad-hoc, self-report questionnaire for any contraindication to tDCS (e.g., metallic implants, epilepsy, pregnancy, severe skin diseases in the area of electrode placement), as well as for past/present neurological or psychiatric disorders (Nitsche et al., 2008). Participants were free from medication (including central nervous system-active drugs) at the time of the experiment and were required to avoid intake of alcohol and caffeinated drinks prior to the experiment. All participants were also screened for depressive symptoms in the past 2 weeks through the Beck Depression Inventory-II (BDI-II) (Beck, Steer, Ball, & Ranieri, 1996a). One participant was excluded due to a BDI-II score of 15, indicating mild depression (Beck, Steer, & Brown, 1996b). None of the participants self-reported past depressive episodes. Thus, the final sample consisted of 38 participants (18 women, mean age ± standard deviation, 22.9 ± 3.7 years).

### Dispositional affect

Dispositional affect was assessed with the Positive and Negative Affect Schedule (PANAS) (Watson et al., 1988), which consists of 20 items describing different feelings and emotions. The items are grouped in positive affect (PA) and negative affect (NA) subscales. The higher the score for each subscale, the higher the level of PA or NA. High PA scores indicate high energy, concentration, and pleasurable engagement, while low PA reflects sadness and lethargy. High NA scores indicate anger, contempt, disgust, guilt, fear, and nervousness, while low NA scores indicate calmness.

Participants rated each item on five points Likert-like scale ranging from 1 (“very slightly or not at all”) to 5 (“extremely”), depending on the extent to which they felt a specific emotion in a precise moment (PANAS- state form) or as a general tendency (PANAS-trait form). We used the PANAS-state form to measure state positive (state PA) and negative affect (state NA) changes before and after tDCS stimulation (see below) and the PANAS-trait form to measure dispositional PA and NA as stable dimensions of the personality (i.e., trait PA, trait NA).

### Emotional dot-probe task

**Fig. 1:**
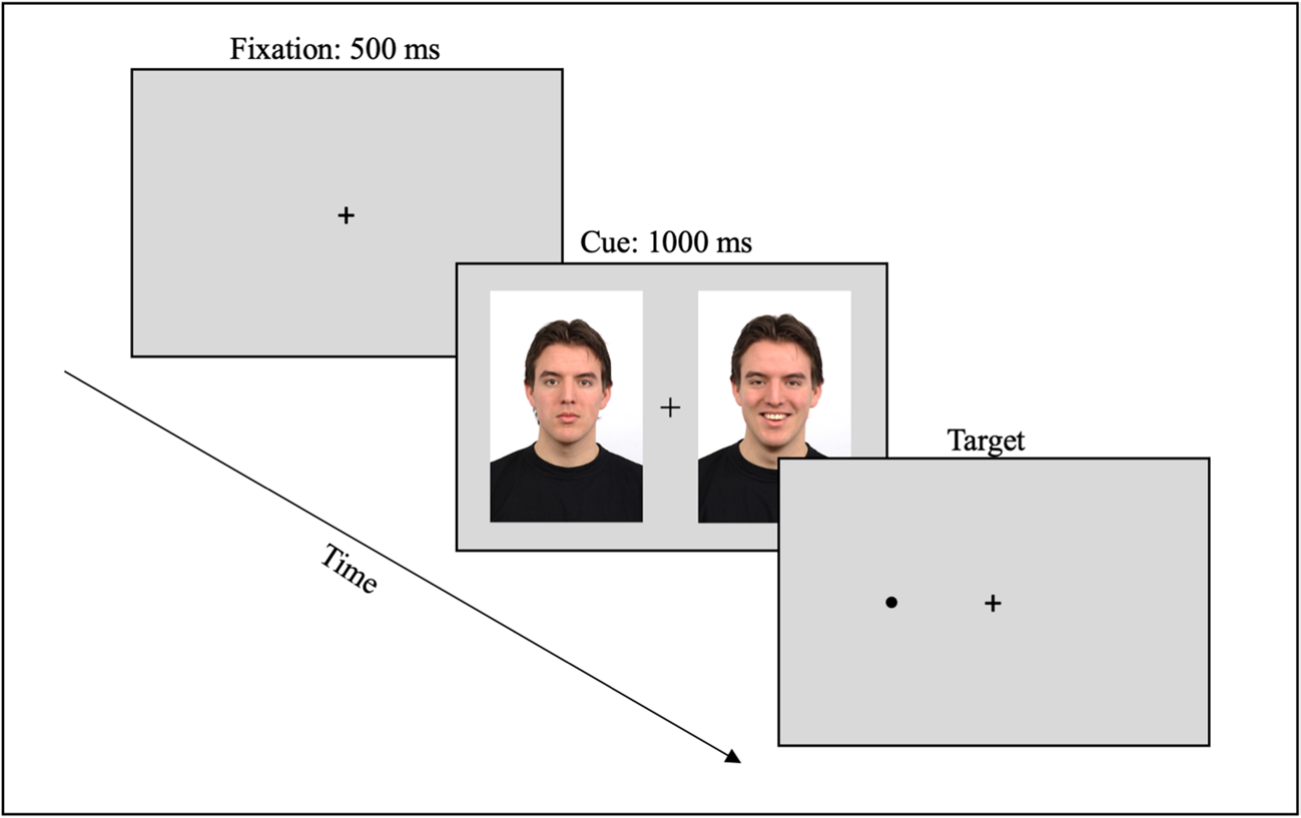
Dot-probe task. In each trial, a fixation cross appeared on the screen for 500 ms; then, emotional (happy, angry, sad) and neutral faces were displayed on either side of the cross. After 1,000 ms, a dot-probe appeared in place of one of the two faces until the participant responded

The dot-probe task was programmed with E-Prime 2.0 software (Psychology Software Tools, Pittsburgh, PA) (Schneider et al., 2002). Stimuli were presented on an IBM-compatible computer and a 15-in color monitor. We used a version of the dot-probe task with faces as emotional or neutral stimuli (Marotta et al., 2020; Marotta et al., 2022; Mather & Carstensen, 2003). A total of 40 stimuli (9 × 10 cm) were selected from the Radboud Faces Database (Langner et al., 2010). The pictures included ten models (5 females) expressing happy, angry, sad, and neutral emotions. Each emotional expression was paired with a neutral expression on the same model. In each pair (i.e., Happy-Neutral, Angry-Neutral, Sad-Neutral), the emotional and neutral faces were positioned one beside the other 13-cm apart, measured from the center. Participants completed the task in a quiet room, sitting on a chair facing a PC monitor at 50 cm in front of them. A keyboard placed centrally to the body midline was used to collect participants’ answers. For each trial, a fixation cross was followed after 500 ms by a face pair (emotional and neutral faces). In half trials, the emotional face appeared on the left and the neutral on the right side of the monitor, and vice versa in the other half of the trials. The faces remained on the screen for 1,000 ms. A dot-probe appeared in place of the emotional or neutral face of the face pair (Fig. 1).

Participants were required to maintain their gaze on the fixation cross until the end of the trial and to press as fast as possible the "K" (using the right index finger) or the "D" (using the left index finger) key on the keyboard according to the position of the dot. The dot remained on the screen until a response was given. Accuracy and reaction times were finally recorded and stored. Participants completed ten practice trials with a neutral-neutral face pair to familiarize themselves with the task and then 120 experimental trials, which consisted of ten trials for each cue condition (Happy-Neutral, Neutral-Happy, Angry-Neutral, Neutral-Angry, Sad-Neutral, Neutral-Sad) and two dot locations (emotional face, neutral face). Face pairs were presented in a randomized order. The total duration of the task was approximately 10 min.

With this experimental paradigm, faster RT to the dot replacing the emotional stimuli hints at a preferential allocation of attention towards emotions, while faster RT to the dot appearing in place of the neutral face denotes attentional avoidance of emotional stimuli (Bradley et al., 1999; Gotlib et al., 2004; Marotta et al., 2020, 2022).

### Transcranial direct current stimulation

**Fig. 2:**
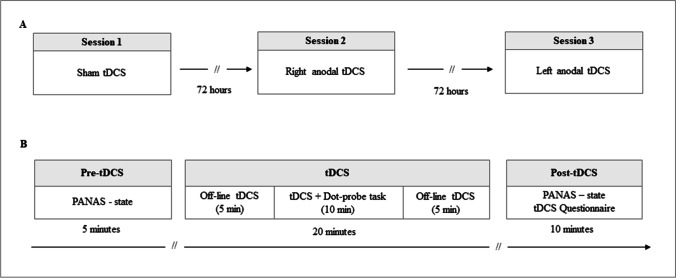
Study flow diagram. **A**) All participants completed three sessions separated by a break of 72 h. The three sessions were similar except for the type of tDCS (sham, right anodal, left anodal) delivered with counterbalanced order across participants. **B**) In each session, before the stimulation (pre-tDCS), participants filled in the PANAS-state form. Then, participants underwent the dot-probe task while receiving tDCS stimulation (lasting 20 minutes). After the stimulation (post-tDCS), participants completed the PANAS-state form again and the tDCS questionnaire

The dot-probe task was performed during three types of tDCS: sham tDCS, anodal tDCS over the right PFC, and anodal tDCS over the left PFC. The three tDCS protocols were performed in three different sessions on 3 days, separated by at least 72 hours, to avoid carry-over effects (Fig. 2).

To prevent any potential bias due to the order of presentation of tDCS protocols, we administered the three types of tDCS (i.e., right-tDCD, left-tDCS, sham-tDCS) with counterbalanced order across participants. In each session, the dot-probe task started 5 minutes after the stimulation protocol onset to ensure a sufficient change in cortical excitability (Peña-Gómez et al., 2011). At the end of each session, participants were asked to fill in a questionnaire assessing the sensations induced by tDCS, and a questionnaire assessing whether they thought tDCS was active, inactive, or were not sure (Fertonani et al., 2015). Participants were debriefed about the purpose of the study and the type of stimulation after the last session.

Direct current stimulation was delivered by BrainStim neurostimulator (E.M.S. Bologna, Italy) through a pair of rubber electrodes (5 × 5 cm) inserted in saline-soaked sponges (0.09% saline solution) and fixed to the participant’s head through two elastic straps.

The tDCS stimulation parameters were the same as in a previous tDCS study on the effect of PFC on emotional processing (Peña-Gómez et al., 2011). For anodal tDCS, the current intensity was set at 1 mA. Stimulation lasted 20 minutes, including 10 sec of ramp-up and 10 sec of ramp-down. For sham tDCS, the electrical current was applied for 30 sec at the beginning and the end of the stimulation period to induce the same cutaneous sensation as the real stimulation but without affecting the activity of the target brain area (Gandiga et al., 2006; Nitsche et al., 2008).

**Fig. 3:**
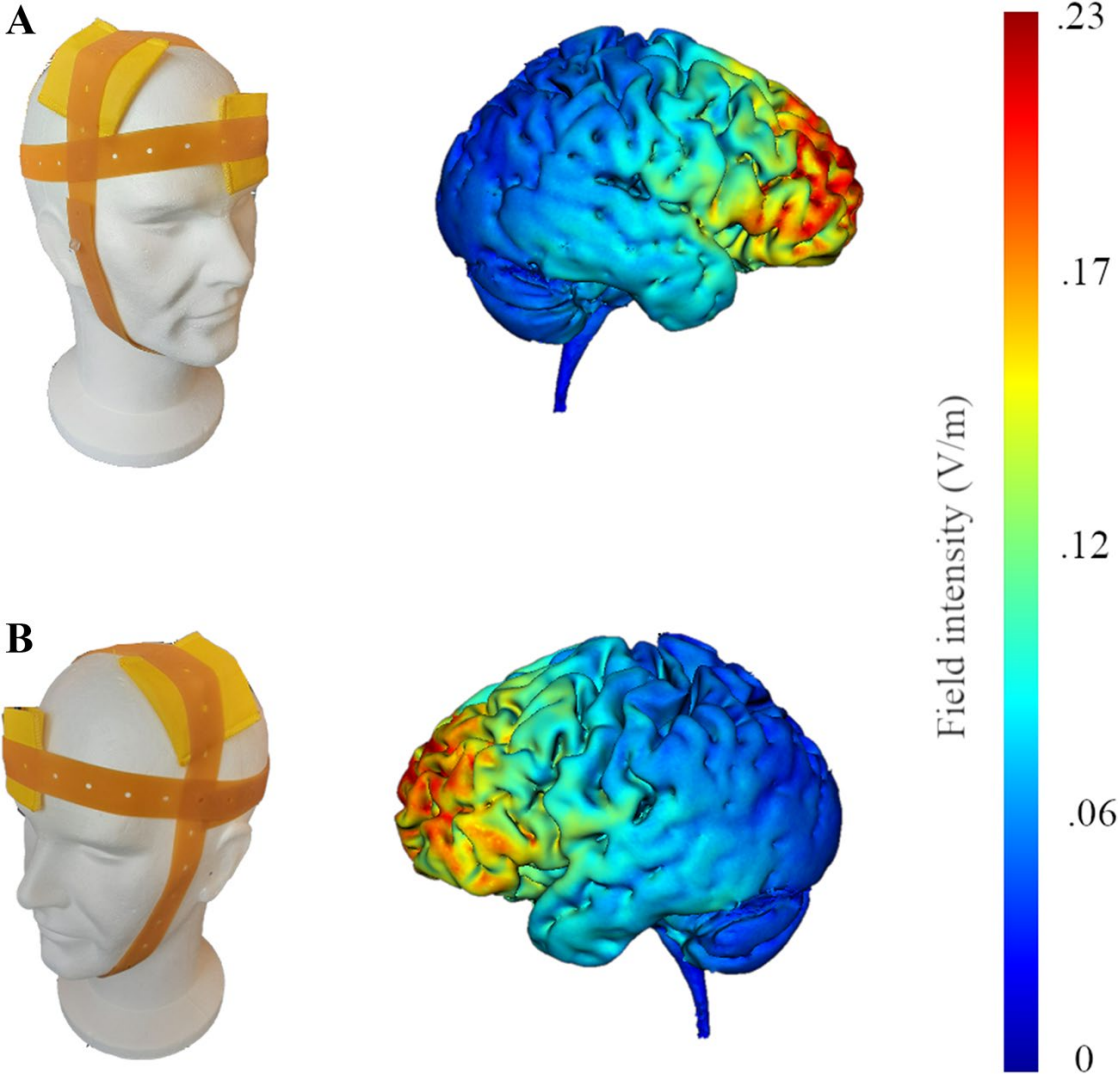
Electrode montage (left panel) and electric field strength (right panel) on a model head for stimulation of the right (**A**) and the left prefrontal cortex (**B**). Estimated electric field strength obtained on a standardized brain by HD-Explore software (HD-Explore 2018, Soterix Medical, NY) shows that the montage used is suitable to stimulate the right and left prefrontal cortex, with some small spread to surrounding brain areas. The red color indicates higher electrical field intensity

According to the EEG international 10-20 system, the anode was placed over F4 to stimulate the right PFC and over F3 to stimulate the left PFC. These scalp locations roughly correspond to the dorsolateral region of the PFC. The cathode was positioned over the contralateral supraorbital area (left or right, respectively, Fig. 3). The electrode placement is in accordance with previous tDCS studies and has shown to produce significant effects on cognitive and emotional processing (Allaert et al., 2019; Dedoncker et al., 2016; Nitsche et al., 2008; Sanchez-Lopez et al., 2018; Vanderhasselt et al., 2013). For the sham-tDCS protocol, the electrode position was the same as in the right-tDCS montage (for half of the participants) or left-tDCS montage (for the other half).

### Data handling

Data were inspected to check for potential outliers, defined as participants who presented mean values at the dot-probe task above or below three standard deviations from the mean. Three participants resulted as outliers and were removed from the analysis.

Reaction times (RT) for correct answers were used to measure participants’ performance at the dot-probe task. Simple RT to the dot were averaged separately for each pair of stimuli (happy/neutral, angry/neutral, sad/neutral) and dot location (emotional face, neutral face), resulting in three emotion-related RT (RT_happy; RT_angry; RT_sad), and three neutral-related RT (RT_neutral_happy_; RT_neutral_angry_; RT_neutral_sad_). We computed an index of attentional bias as follows:

$${AB}_P=\frac{E_P-{NE}_P}{NE_P}$$

where AB_P_ is the attentional bias in the protocol p, E_P_ is the mean RT to the dot appearing in place of the emotional face (RT_happy, RT_angry, RT_sad), and NE_P_ is the mean RT to dot appearing in place of the neutral face (RT_neutral_happy_, RT_neutral_angry_, RT_neutral_sad_). This computation allowed us to normalize the attentional shift in each face pair to the RT_neutral, which is a stable and constant stimulus throughout conditions and participants. Following this procedure, we calculated three different AB scores for each protocol: AB_happy, AB_angry, and AB_sad, with negative and positive values reflecting attention toward or away from the emotional face (Marotta et al., 2022).

### Statistical analysis

Data were analyzed with SPSS Statistics 26 (IBM Corp., 2019) and JASP (JASP team, 2019). As in previous studies (Liuzza et al., 2015), we first performed analyses of Covariance (rmANCOVAs) with Emotion (AB_happy, AB_angry, AB_sad) and tDCS protocol (sham-tDCS, right-tDCS, left-tDCS) as within-subject factors and PANAS-trait (trait NA or trait PA) as covariate. This analysis allowed us to explore the role of dispositional negative and positive affect in the interplay between tDCS over the PFC and attentional bias to emotions. According to the literature, PANAS-trait NA and PA represent two independent constructs (Garcia et al., 2015), and therefore, they were entered as covariates into separate ANCOVAs. Spearman correlations between PANAS-trait NA and PA subscales confirmed the lack of association between trait NA and trait PA in our sample (rho = −0.237, *p* = 0.185). In case of significant interaction between the covariate (trait PA or trait NA) and the main factors, subsequent analyses were conducted by splitting the sample according to the median value and inserting the group (higher vs. lower) as between-subject factor in the ANOVA. In these analyses, the factor Session order was included as covariate to control for a potential effect of this factor on our findings. Paired and independent t-test was used for post-hoc analysis. Additionally, to check whether the level of trait NA or trait PA differed between groups, we compared PANAS trait scores between groups by means of Mann-Whitney *U* test.

To determine whether AB was oriented toward or away from the emotional stimuli, AB scores were compared against zero (with 0 indicating unbiased attentional response) using one-sample *t*-test and Bayesian one-sample *t*-tests (Bradley et al., 1999; Joormann & Gotlib, 2007; Marotta et al., 2020, 2022).

To explore the potential effect of tDCS on the participants’ affective state, PANAS-state scores obtained before and after tDCS stimulation were compared by means of the Wilcoxon signed-rank test, due to the nature of the data. Separate Friedman tests were also performed to assess differences across tDCS protocols for each time (pre-tDCS, post-tDCS). Finally, Spearman coefficient of correlation was used to assess any relation between PANAS-trait and PANAS-state scores in all sessions and time.

Effect size was estimated with partial eta squared for ANOVA (η_P_^2^) (Keppel, 1991), and Cohen’s d (d) for paired *t*-tests (Lakens, 2013). Statistical significance was set at *p* < 0.05. Bonferroni corrections were applied when necessary.

## Results

### Attentional bias

**Fig. 4:**
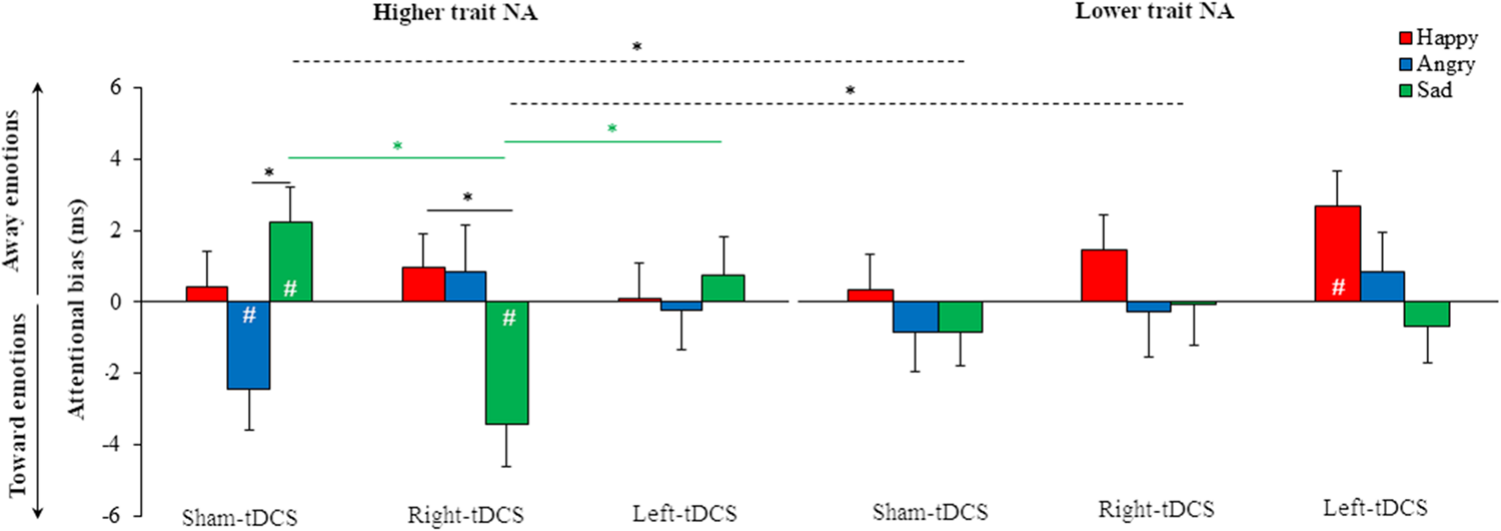
Attentional bias scores for happy (red), angry (blue), and sad faces (green) for higher trait NA (left panel) and lower trait NA individuals (right panel). Dashed lines and solid lines indicate significant differences between groups and across conditions, respectively. Asterisks indicate *p* < 0.05. The hashtags show significant comparisons against zero (*p* < 0.05). Bars represent standard errors

Trait PA as a covariate did not yield statistically significant results (*p* > 0.134), whereas the interaction between Emotion, tDCS protocol, and trait NA as a covariate approached statistical significance (F_(4,132)_ = 2.419, *p* = 0.052, η_P_^2^ = 0.068). To explore this interaction more deeply, we split the sample according to the median of the NA score (median = 21) (Liuzza et al., 2015). Participants with trait NA scores > 21 were assigned to the higher trait NA group (n = 16, 8 men and 8 women, mean age ± SD, 22.125 ± 2.553), whereas those with trait NA scores < 21 were assigned to the lower trait NA group (n = 17, 9 men and 8 women, mean age ± SD, 23.118 ± 3.723). Two participants were removed because of trait NA scores equal to the median. The Mann-Whitney *U* test showed significantly higher level of trait NA in the higher trait NA group (mean trait NA ± SD, 27.813 ±5.218) than in the lower trait NA group (16.353 ± 2.914) (z = −4.910, *p* < 0.0001). We ran a mixed-model analysis of variance (ANOVA) with trait NA group (higher trait NA vs. lower trait NA) as between-subjects factor and Emotion (AB_happy, AB_angry, AB_sad) and tDCS protocol (sham-tDCS, right-tDCS, and left-tDCS) as within-subjects factors. The analysis revealed a significant main effect of the Emotion (F_(2,62)_ = 3.436, *p* = 0.038, η_P_^2^ = 0.100) and a Group × Emotion × tDCS protocol interaction (F_(4, 124)_ = 3.037, *p* = 0.020, η_P_^2^ = 0.089). Post-hoc comparisons showed that with sham-tDCS, higher trait NA participants presented an opposite pattern for AB_sad (mean ± standard error, 2.234 ± 0.979) and AB_angry (−2.449 ± 1.137) (*p* = 0.006, d = −0.853). Whereas AB_sad was significantly greater than 0, indicating a bias away from sad faces (t_(15)_ = 2.128, *p* = 0.050, d = 0.532), AB_angry was significantly lower than 0, indicating a bias toward angry faces (t_(15)_ = −2.152, *p* = 0.048, d = −0.538) (Fig. 4). These findings were corroborated by the Bayesian factor analysis, which confirmed the alternative hypothesis for AB_sad (BF_10_ = 1.495) and AB_angry (BF_10_ = 1.549).

Right-tDCS partially reversed this pattern of results in higher trait NA participants, in that AB_sad (−3.45 ± 4.24) was significantly lower than 0 (t_(15)_ = −3.258, *p* = 0.005, d = −0.814), indicating an attentional bias toward sad faces, whereas AB_angry did not differ from 0 (Fig. 4). Bayesian factor analysis confirmed the alternative hypothesis for AB_sad (BF_10_ = 9.272) and the null hypothesis for AB_angry (BF_10_ = 0.316). Moreover, in higher trait NA participants, AB_sad was significantly lower than AB_happy (0.942 ± 0.981) (*p* = 0.019, d = −0.717). Of note, no difference against 0 was found for AB_happy, indicating no bias for happy faces. Additionally, right-tDCS determined more negative AB_sad compared with both sham-tDCS (*p* = 0.002, d = −1.133) and left-tDCS (0.754 ± 1.069, *p* = 0.021, d = −0.660), suggesting a critical role of the right PFC in attentional bias toward sad faces in higher trait NA participants (Fig. 4).

Regarding lower trait NA participants, the main analysis did not reveal any statistically significant factor or interaction (for all comparisons, *p* > 0.337), but only a significant difference form 0 for AB_happy (2.626 ± 0.965) (t_(16)_ = 2.224, *p* = 0.041, d = 0.539), indicating a bias away from happy faces selectively in left-tDCS protocol (Fig. 4). This result was further confirmed by the Bayesian factor analysis, which supported the alternative hypothesis for AB_happy (BF_10_ = 1.723).

By comparing the two groups, we found differences between higher trait NA and lower trait NA with regards to the AB_sad in the sham-tDCS (*p* = 0.031, d = −0.785) and in the right-tDCS (*p* = 0.049, d = 0.714). In the sham-tDCS, AB_sad was higher in higher trait NA than in lower trait NA participants (−0.841 ± 0.950). Right-tDCS reversed this pattern, determining a significantly lower AB_sad in higher trait NA individuals than in the lower trait NA individuals (0.095 ± 1.140, *p* = 0.049, d = 0.714) (Fig. 4).

Session order as covariate did not interact with any other factor (all *p* > 0.230), thus ruling out an effect of the order of the sessions on our results. The interaction Group × tDCS protocol × Emotion was significant even when including Session order as covariate (F_(4,120)_ = 3.173, *p* = 0.016), thus hinting at the robustness of our main findings.

### PANAS state

PANAS-state scores were not different between pre- and post- tDCS stimulation in all sessions (all *p* > 0.124). The Friedman test comparing PANAS-state scores across sessions did not yield statistical results (all *p* > 0.356), thus excluding a significant effect of tDCS on affective state.

### Correlation between PANAS trait and state

The PANAS trait NA significantly correlated with the PANAS state NA in all tDCS protocols and time (pre, post) (all *p* < 0.041), except for pre-Right-tDCS and post-Left-tDCS. The PANAS trait PA significantly correlated with the PANAS state PA in all tDCS protocols and time (all *p* < 0.001). These findings are in line with previous studies suggesting that trait and state features of NA and PA are related (Li et al., 2022).

## Discussion

This study investigated the interplay between dispositional affect and the PFC in attentional bias to emotions by applying sham and anodal tDCS over the left and right DLFPC during a dot-probe task. Despite the ample body of evidence on the effect of tDCS on PFC in modulating emotional processes (Boggio et al., 2009; Clarke et al., 2020; Sanchez-Lopez et al., 2018; Vanderhasselt et al., 2013), only one tDCS study has previously considered the role of personality traits on the neural underpinnings of emotional processing (Peña-Gómez et al., 2011). Interestingly, the authors found that introverts were more permeable to the tDCS effect than extraverts. Indeed, anodal tDCS selectively reduced the perceived valence of emotionally negative pictures in the former but not in the latter. Because only the left PFC was stimulated, potential trait-related hemispheric differences in emotional processing remained unknown. Moreover, the use of subjective measures, such as ad-hoc scales to judge the valence of emotional stimuli (Peña-Gómez et al., 2011), does not allow to uncover implicit emotional processes. The current study brings new evidence on this line, by exploring trait-related neural bases of emotional processes through a more comprehensive methodological approach. First, we focused on dispositional affect, which is a leading personality trait in shaping emotional information processing. Second, we explored trait-related hemispheric differences by applying tDCS on both the left and the right PFC. Third, we tackled implicit emotional processes (i.e., attentional bias) by means of the dot-probe task. In line with our hypothesis, stimulation of the right or the left PFC influenced the attentional bias to emotional stimuli differently, depending on individual levels in trait NA. Conversely, we did not find any modulation of attentional bias due to trait PA, suggesting that NA, more than PA, could be associated with heightened sensitivity to emotional stimuli. In fact, individuals with higher trait NA struggle to ignore emotional information (Crocker et al., 2012). Moreover, neuroticism and anxiety, two NA-related traits, have been linked to heightened alertness to emotional stimuli (Andric et al., 2016; Doty et al., 2013; Haas et al., 2007, 2008).

Going deeper into the role of dispositional NA on the attentional bias to emotions, a different pattern of results emerged in individuals with higher and lower levels of dispositional NA, even in the sham tDCS session. Being sham tDCS inactive, the differences in attentional bias to emotions between these two groups can be ascribed reasonably to the different levels of dispositional NA. More precisely, while lower trait NA individuals did not show any attentional bias for the tested emotions, higher trait NA individuals had a clear attentional bias toward angry faces and away from sad faces. Of note, heightened attentional bias toward or away from negative emotional stimuli is considered to be dysfunctional, because it acts as a risk and maintaining factor of various forms of psychopathology, such as depression and anxiety (Bradley et al., 1999; Clarke et al., 2014, 2020; Gotlib et al., 2004). The heightened attentional bias toward angry faces indicates a difficulty in disengaging attention from threatening emotional stimuli, as extensively demonstrated by previous studies (Harrewijn et al., 2021; Kircanski et al., 2018; Mekawi et al., 2020; Oehlberg et al., 2012). Conversely, the attentional bias away from sad faces could indicate that higher trait NA individuals tend to avoid sadness. Although this interpretation should be taken with caution, it finds support from studies showing avoidance of sad faces in individuals with trait NA (Oehlberg et al., 2012). Overall, the findings of the sham session suggest that different levels of trait NA (higher vs. lower) influence attentional bias to negative emotions (Oehlberg et al., 2012; Onie & Most, 2017).

By taking into account the findings obtained with active tDCS, and discussed below, this result could be explained by hypothesizing a different baseline level in the activity of the PFC in higher and lower trait NA individuals that could have induced, in turn, a different response to emotional stimuli. Along this line, the activity of the posterior PFC to emotional stimuli was found to be reduced in individuals with higher trait NA, leading to impaired top-down attentional control of negative emotions (Crocker et al., 2012). Furthermore, reduced activity of the right PFC has been found in patients with mood disorders, in whom high trait NA is a key feature (Drevets, 1998; Mayberg, 2003). Overall, it seems that reduced activity of the PFC (together with alterations in other systems, such as the limbic system) leads to dysfunctional attentional bias to negative emotional information (Bishop, 2007), particularly in individuals with higher levels of trait NA. Hence, we hypothesize that the dysfunctional attentional bias to negative emotions (angry and sad faces) observed in higher trait NA individuals in the sham tDCS session could be due to reduced activation of the PFC. Of course, this speculation needs to be proven by directly recording the activity of this brain region.

Interestingly, anodal tDCS over the right PFC determined a reversed pattern of results higher trait NA individuals, which seems to support our hypothesis. In detail, tDCS over the right PFC abolished the attentional bias toward angry faces and enhanced the attentional bias toward sad faces. This finding is consistent with the evidence that heightened activity of the right PFC is associated with reduced attentional engagement toward angry faces (De Readt et al., 2010) and that increasing the activity of the right PFC with high-frequency rTMS impairs the ability of healthy individuals to inhibit the processing of sad faces (Leyman et al., 2009). We speculate that anodal tDCS over the right PFC could have increased the activity of this brain area, thus reducing attention allocation toward angry faces and impairing the inhibitory processing of sadness (inducing a bias toward sad faces) (Leyman et al., 2009). The fact that this pattern was specifically observed in individuals with higher trait NA indicates that the right PFC could be more susceptible to the effects of stimulation in higher trait NA than in lower trait NA individuals, especially when facing negative emotions. The right PFC seems to be mainly involved in processing negative emotional cues (Baeken et al., 2010). Therefore, we could hypothesize that individuals with higher levels of trait NA are more prone to modulation of this area in the context of negative emotions.

Stimulation of the left PFC abolished any attentional bias to emotions in higher trait NA individuals, thus hinting at a potential balancing role of this area. This is in line with previous studies in healthy volunteers which demonstrated that anodal tDCS over the left PFC has beneficial effects on emotion regulation, being associated with a decreased emotional reactivity towards negative stimuli (Boggio et al., 2009; Brunoni et al., 2013; Clarke et al., 2020; Peña-Gómez et al., 2011). Moreover, tDCS over the left PFC reduced attentional interference of positive and negative emotional distractors in a Stroop task in depressed individuals (Brunoni et al., 2014) and decreased attentional bias to fearful emotional information in healthy and socially anxious individuals (Heeren et al., 2017; Ironside et al., 2016). We hypothesize that anodal tDCS over the left PFC could have increased the activity of this brain region, thus enhancing the top-down regulation of emotions in higher trait NA individuals.

Our findings can be explained within the framework of frontal asymmetry theories, suggesting that dispositional negative affect and poor regulation of negative emotions are associated to higher right than left prefrontal activity (Jackson et al., 2003; Thibodeau et al., 2006). Of note, functional hemispheric imbalance of the PFC has been found in patients with mood disorders, which are characterized by a pattern of relatively less left than right resting frontal activity (Disner et al., 2011; Grimm et al., 2008). Consistent with this view, enhancing the activity of the left PFC by means of neuromodulation techniques was shown to decrease the attentional bias to negative emotions in clinically anxious and depressed individuals (Heeren et al., 2017; Ironside et al., 2016). The current study suggests that such an effect can be obtained also in healthy individuals with high trait NA, hinting at a possible strategy to favor top-down control of attention toward negative emotional stimuli in individuals at risk to develop mood disorders.

Lower trait NA individuals, instead, had a less attentional bias to emotion and were less sensitive to the effects of tDCS. More precisely, lower trait NA individuals showed unbiased attention in the sham session, thus excluding any influence of emotional stimuli on attentional processing. Surprisingly, when anodal tDCS was applied over the left PFC, an attentional bias away from happy faces was found in this group. This result could hint at a heightened cognitive control of positive stimuli induced by the stimulation of the left PFC. In other words, anodal tDCS over the left PFC could have facilitated attentional avoidance of positive stimuli as a control strategy in these individuals. In line with this, a previous study found enhanced cognitive control in response inhibition to happy faces after anodal tDCS of the left PFC (Vanderhasselt et al., 2013). It could be also argued that when neutral faces were presented together with happy faces, the former could have acquired a sort of aversive or threatening valence. However, because in the other face-pairs (sad-neutral, hangry-neutral) we did not find a similar effect, we would exclude this hypothesis. Future studies are needed to better clarify the role of left PFC in attentional processing of neutral compared to positive emotions.

Overall, our findings suggest that dispositional affect might determine different neural responses to emotional stimuli (Calder et al., 2011; Canli, 2004; Crocker et al., 2012). As we know, the cortical activation state at the time of brain stimulation exerts an influence on the effect of the stimulation itself (Silvanto et al., 2008). In other words, the effects that an external stimulus (like the electrical current) exerts on a brain region are defined not only by the physical properties of the stimulus but also by the baseline activation of the brain region. This effect has been called “state-dependency.” It is tempting to consider negative affect as a dispositional factor inducing a particular brain state (e.g., hypoactivation of the right and left PFC in higher trait NA individuals). More broadly, we could interpret our findings in light of the interaction between a brain state associated with the level of negative affect and the electrical modulation of a brain area potentially involved in emotion regulation.

Finally, we did not find significant effect of tDCS on the emotional states as measured by the PANAS state. This result is in line with previous studies showing that tDCS did not influence affective state (Mondino et al., 2015; Vanderhasselt et al., 2013), and support the hypothesis that the effect of tDCS on attentional bias to emotion is specifically modulated by dispositional affect rather than by individuals’ affective state.

This study has some limitations. First, the dot-probe task consisted of a restricted set of emotional stimuli, limiting the generalizability of our findings to the real-world context where individuals are presented with social cues varying in emotional valence and salience. Moreover, this task has been found to present a poor test-retest reliability in nonclinical studies (Schmukle, 2005). Although this could mean that changes in attentional bias might be due to the instability of measurements across sessions, the evidence of a specific pattern of results for different types of tDCS protocols suggests that our experimental design was able to capture a clear effect of tDCS on attentional bias to emotions. Second, we did not measure anxiety in our study. However, because anxiety appears to be associated with negative affect and with attentional bias to negative emotions (Böhnke et al., 2014; Bradley et al., 1998, 1999; Joiner Jr. & Lonigan, 2000; Watson et al., 1988), it could be considered a mediating factor to be included in future studies. Third, although the electric field of tDCS was maximally localized over the region of interest, we cannot exclude that surrounding brain regions also were stimulated (Datta et al., 2009). Future studies with other techniques (such as TMS combined with fMRI) may help to gain more precise knowledge. Finally, to deeply explore the role of negative affect, we split the sample into two groups: higher trait NA and lower trait NA. Despite this approach being effective in providing fine-tuned evidence on the role of negative affect, it could be argued that the reduced sample size of the two groups has undermined the statistical power of our findings. However, we think that this hypothesis can be excluded. Indeed, we obtained medium to large effect sizes for significant results, thus supporting the strength of our findings.

These limitations notwithstanding, the present study adds new insights to the growing body of evidence on the impact of personality traits in modulating the role of PFC in emotional processing. We demonstrated for the first time that negative dispositional affect might strongly influence the effect of tDCS over the PFC on attentional bias to emotional stimuli, providing further evidence on the impact of personality traits in modulating individuals' responses to tDCS (Peña-Gómez et al., 2011; Reyes et al., 2021). Meanwhile, by applying anodal tDCS over the right and the left PFC, we provided further support to the hemispheric specialization of the PFC in the top-down regulation of attention to emotions.

This study might inspire future research to improve attentional regulation of emotional processes in individuals with high trait negative affect, thus helping to promote psychological well-being and reduce the risk of developing mood disorders.

## Data Availability

Not applicable.

## Data availability

The raw data are available from the corresponding author upon request.
